# Simplified Clinical Prediction Scores to Target Viral Load Testing in Adults with Suspected First Line Treatment Failure in Phnom Penh, Cambodia

**DOI:** 10.1371/journal.pone.0087879

**Published:** 2014-02-04

**Authors:** Johan van Griensven, Vichet Phan, Sopheak Thai, Olivier Koole, Lutgarde Lynen

**Affiliations:** 1 Sihanouk Hospital Center of HOPE, Phnom Penh, Cambodia; 2 Institute of Tropical Medicine, Antwerp, Belgium; 3 London School of Hygiene and Tropical Medicine, London, United Kingdom; Boston University, United States of America

## Abstract

**Background:**

For settings with limited laboratory capacity, 2013 World Health Organization (WHO) guidelines recommend targeted HIV-1 viral load (VL) testing to identify virological failure. We previously developed and validated a clinical prediction score (CPS) for targeted VL testing, relying on clinical, adherence and laboratory data. While outperforming the WHO failure criteria, it required substantial calculation and review of all previous laboratory tests. In response, we developed four simplified, less error-prone and broadly applicable CPS versions that can be done ‘on the spot’.

**Methodology/Principal:**

Findings From May 2010 to June 2011, we validated the original CPS in a non-governmental hospital in Phnom Penh, Cambodia applying the CPS to adults on first-line treatment >1 year. Virological failure was defined as a single VL >1000 copies/ml. The four CPSs included CPS1 with ‘current CD4 count’ instead of %-decline-from-peak CD4; CPS2 with hemoglobin measurements removed; CPS3 having ‘decrease in CD4 count below baseline value’ removed; CPS4 was purely clinical. Score development relied on the Spiegelhalter/Knill-Jones method. Variables independently associated with virological failure with a likelihood ratio ≥1.5 or ≤0.67 were retained. CPS performance was evaluated based on the area-under-the-ROC-curve (AUROC) and 95% confidence intervals (CI). The CPSs were validated in an independent dataset. A total of 1490 individuals (56.6% female, median age: 38 years (interquartile range (IQR 33–44)); median baseline CD4 count: 94 cells/µL (IQR 28–205), median time on antiretroviral therapy 3.6 years (IQR 2.1–5.1)), were included. Forty-five 45 (3.0%) individuals had virological failure. CPS1 yielded an AUROC of 0.69 (95% CI: 0.62–0.75) in validation, CPS2 an AUROC of 0.68 (95% CI: 0.62–0.74), and CPS3, an AUROC of 0.67 (95% CI: 0.61–0.73). The purely clinical CPS4 performed poorly (AUROC-0.59; 95% CI: 0.53–0.65).

**Conclusions:**

Simplified CPSs retained acceptable accuracy as long as current CD4 count testing was included. Ease of field application and field accuracy remains to be defined.

## Introduction

Scaling-up of antiretroviral treatment (ART) is currently ongoing in low and middle income countries (LMIC), aiming to initiate 15 million individuals on ART by 2015 [1]. One of the key challenges for program managers and policy makers in these countries is how to monitor these individuals for treatment failure, considering the often limited financial resources at hand [2].

Routine viral load (VL) testing is now recommended by the World Health Organization (WHO) [3] but, with currently available technologies, comes at a high cost and is technically demanding. Thus, it will still take many years before this will be readily available in routine program settings in many LIMC [4], [5]. Instead, for settings with limited VL testing capacity, the 2013 WHO guidelines recommend targeted VL testing [3].

Targeted VL testing, whereby VL testing is done only in individuals meeting failure criteria, aims to avoid unnecessary and costly switches to second line treatment for patients with false-positive ‘screening’ tests [2]. Effective implementation of such a strategy requires accurate and evidence-based tools to target VL testing. Whereas most programs have been applying WHO clinical and immunological failure criteria [6], studies have consistently demonstrated the low sensitivity and specificity of these criteria [2], [7].

We previously developed a clinical prediction score (CPS) for virological failure integrating clinical, adherence and laboratory data [8]. At the same time, we constructed an algorithm combining the CPS with targeted VL testing (in patients with a CPS ≥2). The score performed substantially better than the WHO failure criteria, and performed well in internal validation (Cambodia) and external validation (Uganda) [9], [10]. Experience from Lesotho provided additional support for its use in patients who were identified based on WHO immunological and clinical criteria as treatment failure [11]. A few other CPSs have been developed for the same purpose, but they have either not been validated, or performed poorly during validation [9], [12], [13].

Two limitations of the original CPS were identified during the validation study in Cambodia [10]. Firstly, frequent errors were made when physicians applied the score, which affected the CPS performance. Errors in scoring of the individual items were most commonly seen for items like the percentage decrease from peak CD4 cell counts or the decline in hemoglobin values since these items require calculation, and availability and review of all previous laboratory results. Secondly, the reliance on regular laboratory monitoring of CD4 count and hemoglobin values limit implementation of the original CPS in settings where such tests are not routinely performed. In response to these limitations, we derived and validated several simplified versions of the original clinical score, aiming to make the tool less error-prone, easier to apply and more broadly applicable.

## Methods

### Study Setting

Sihanouk Hospital Center of HOPE (SHCH) is a non-governmental hospital in Phnom Penh Cambodia. Since 2003, the hospital has provided ART at no cost as part of the national program. Patients were initiated and treated according to WHO recommendations [6], [14]. First line treatment consisted of a generic combination of stavudine, lamuvidine and nevirapine. Zidovudine and efavirenz was used in case of contraindications. The revised 2010 guidelines were implemented in May 2010. Patients were seen at regular intervals for clinical and laboratory monitoring and adherence assessment. All care was provided by physicians. More information on the program has been published before [15]–[17].

### Validation Study of the Original CPS

Details of the original validation study have been previously reported [10]. In brief, we conducted a cross-sectional study within an established ART cohort in Cambodia between May 10, 2010 and June 3, 2011, including all consenting adults on a non-nucleoside containing (standard) first-line regimen for a minimum of one year (n = 1490). The original score – derived in this hospital - contained eight items: 1) CD4 decline from peak >25%, 2) CD4 decline from peak >50%, 3) hemoglobin drop≥1 g/dL over the last six months, 4) current CD4 below baseline, 5) CD4 count <100 cells/µL at 12 month of ART, 6) papular pruritic eruption (PPE), 7) treatment adherence <95% (measured using a visual analogue score); 8) ART-experience [8]. Summing the predictor scores of the individual’s risk factors yielded the total predictor score for each patient. Virological failure was defined as a VL above 1000 copies/ml at the cross-sectional survey. The overall test performance was assessed by calculating the area under the receiver-operating characteristic curve (AUROC).

### Development and Validation of Simplified CPS

The following simplified CPSs were evaluated with a progressive increase in simplification by removing: 1) the ‘decline from peak CD4 count values’ and only using ‘current’ CD4 count (CPS1) - ie the value of the CD4 count at the time of assessment for virological failure; 2) hemoglobin as item in the CPS (CPS2); 3) ‘decrease in CD4 count below baseline value’ as item in the CPS (CPS3); and 4) all laboratory items from the CPS (CPS4).

The same methodology for score development was used as for the original score derivation [8]. In brief, we used the Spiegelhalter and Knill-Jones method adapted by Berkley et al [18]–[20]. Continuous variables were dichotomized as guided by ROC curves. Variables independently associated with virological failure with a likelihood ratio (LHR) ≥1.5 or ≤0.67 were retained. The score of each predictor was calculated as the natural logarithm of the adjusted LHR, rounded to the nearest integer. Summing the predictor scores of the individual’s risk factors yielded the total predictor score for each patient. For simplification, the score was recoded by setting the reference level for each predictor to zero [20]. The performance of the different CPSs was based on the AUROC and 95% confidence intervals (CIs). A CPS was considered clinically useful if, during validation, the lower limit of the 95% CI was 0.6 or above. In addition, we calculated the sensitivity, specificity, positive and negative predictive values, the positive and negative likelihood ratio’s and proportion of individuals that would require VL testing at the different cut-off values of each CPS. The simplified scores were developed using the same dataset of the study mentioned above, conducted in 2010–11. The different CPSs were validated in an independent dataset from the same hospital (2005–2007), as reported before [8]. In this study, patients were enrolled following the same inclusion criteria as the 2010–2011 study mentioned above (Table 1). Confidence intervals for the validated AUROCs were calculated using robust standard errors.

**Table 1:** Datasets used for the development and validation of the simplified prediction score to identify virological failure.

	Derivation dataset^a^	Validation dataseta,b
	2010–2011	2005–2007
	1490 individuals	1803 episodes
Age (years) at baseline, median (IQR)	35 (30–41)	34 (18–68)
Male sex, n (%)	646 (43.4)	925 (51.3)
Years on ART at viral load assessment, median (IQR)	3.6 (2.1–5.1)	1.5 (1.0–2.0)
Virological failure - n (%)	45 (3.0)	77 (4.8)
Initial ART regimen		
D4T-3TC-NVP	1056 (70.9)	1369 (75.9)
D4T-3TC-EFV	343 (23.0)	261 (14.5)
AZT-3TC-NVP	62 (4.2)	106 (5.9)
AZT-3TC-EFV	24 (1.6)	63 (3.5)
Other	5 (0.4)	4 (0.2)
CD4 count, cells/µL, median (IQR)		
Baseline (ART initiation)	94 (28–205)	54 (15–147)
At viral load assessment	379 (265–507)	281 (187–398)

a Inclusion criteria: consecutive enrolment of adult patients (≥18 years old) in follow-up at the Sihanouk Hospital Center of Hope on first-line ART for at least 12 months; viral load was systematically performed for study purpose; virological failure was defined as a single measurement >1000 copies/ml;

b more than one episodes per individual.

ART: antiretroviral treatment; AZT: zidovudine; D4T: stavudine; 3TC: lamivudine; NVP: nevirapine; EFV: efavirenz; IQR: interquartile range.

### Ethical Considerations

The VL validation study was approved by the National Ethics Committee for Health Research, Phnom Penh, Cambodia; the institutional review board (IRB) of the Institute of Tropical Medicine, Antwerp, Belgium; and the Ethics Committee of the University Hospital of Antwerp, Belgium. All study participants provided written consent.

## Results

A total of 1490 adults on first-line ART for at least one year were included. As previously described, median time on ART at the time of evaluation of virological treatment response was 3.6 (IQR 2.5–5.1) years. The median age at study inclusion was 38 (IQR 33–44) years and the median CD4 count was 379 (IQR 265–507) cells/µL. Approximately half (56.7%) were female and >90% were on a zidovudine or stavudine-based regimen at the time of evaluation. A total of 45 patients (3.0%) were detected with virological failure.

In a first step (CPS1), we removed CD4 decline from peak value, since it requires substantial calculation and complete on treatment CD4 count data, but retained ‘current CD4 count’. The AUROC of CPS1 was 0.78 (95% CI 0.70–0.85) in derivation and 0.69 (95% CI 0.62–0.75) in validation (Table 2). With the WHO 2013 guidelines arguing against routine hemoglobin monitoring - even for patients using zidovudine - hemoglobin measurement was additionally removed in CPS2. The validated AUROC decreased to 0.68 (95% CI 0.62–0.74). To obtain a tool devoid of any requirement of previous laboratory results (hence only relying on “current” lab tests), comparison with baseline CD4 count was removed in CPS3. Only a slight decrease in AUROC was observed. This provided a system essentially relying on basic clinical information collected during routine patient assessment: clinical evaluation (WHO T-stage, PPE, treatment information (ART-experience and ART adherence) and CD4 response at the time of assessment. No laboratory monitoring was included in CPS4. However, this tool performed poorly (AUROC 0.59 in validation). The sensitivity, specificity, positive predictive value and percentage of individuals requiring a VL at various score cut-off values of the different CPS is given in Table 3, applying the CPS in an independent dataset.

**Table 2:** Content and diagnostic performance of the simplified clinical prediction scores to identify virological failure^a^.

	CPS 1	CPS 2	CPS 3	CPS 4	
	No CD4 peak values	No hemoglobin	No CD4 baseline	No lab investigations	
**AUROC derivation**	0.78	0.75	0.75	0.63	
**(95% CI); (n = 1490)**	(0.70–0.85)	(0.68–0.83)	(0.67–0.82)	(0.56–0.70)	
**AUROC validation**	0.69	0.68	0.67	0.59	
**(95% CI); (n = 1803)**	(0.62–0.75)	(0.62–0.74)	(0.61–0.73)	(0.53–0.65)	
**Items**					
CD4 decline from peak >25%	Not included	Not included	Not included	Not included	
CD4 decline from peak >50%	Not included	Not included	Not included	Not included	
Hemoglobin drop≥1 g/dL^b^	+1	Not included	Not included	Not included	
Current CD4 below baseline	+1	+1	Not included	Not included	
Current CD4<250 cells/µL	+2	+2	+2	Not included	
WHO T-stage ¾^b^	+1	+1	+1	+1	
PPE^b^	+2	+2	+2	+2	
Adherence (VAS) <95%^c^	+1	+1	+2	+2	
ART-experience	+2	+1	+1	+1	

ART: antiretroviral treatment; AUROC: area under the receiver-operating characteristic curve; CPS: clinical prediction score; PPE: papular pruritic eruption; VAS: visual analogue scale.

a differences between CPSs in the score of some predictors relate to the fact that the weight of each remaining predictor was recalculated after removing of specific predictors (*eg* after removing hemoglobin in CPS2 vs CPS1).

b over a period of six months prior to viral load measurement.

c over a period of one month prior to viral load measurement.

**Table 3:** Diagnostic performance at different cut-offs of the clinical prediction scores to identify virological failure using an independent dataset.

Score	Sensitivity	Specificity	PPV	% VL done	LHR(+)	LHR(−)
***CPS 1***						
** ≥1**	80.5	36.5	6.0	64.3	1.27	0.54
** ≥2**	73.6	50.3	7.0	50.8	1.48	0.52
** ≥3**	49.4	80.4	11.3	21.1	2.52	0.63
** ≥4**	34.5	92.1	18.1	9.2	4.35	0.71
***CPS 2***						
** ≥1**	79.1	41.0	6.4	60.0	1.34	0.50
** ≥2**	72.4	54.8	7.5	46.5	1.60	0.50
** ≥3**	41.4	84.7	12.1	16.5	2.71	0.69
** ≥4**	24.1	95.9	23.1	5.0	5.92	0.79
***CPS 3***						
** ≥1**	78.2	41.3	6.3	60.0	1.33	0.53
** ≥2**	72.4	54.8	7.5	46.5	1.6	0.50
** ≥3**	36.8	85.7	11.6	15.4	2.58	0.74
** ≥4**	21.8	96.3	22.9	4.6	5.86	0.81
***CPS 4***						
** ≥1**	43.7	70.0	6.9	30.6	1.46	0.80
** ≥2**	25.3	91.5	13.2	9.3	2.99	0.82
** ≥3**	9.2	97.8	17.8	2.5	4.26	0.93
** ≥4**	1.1	99.9	33.3	0.2	9.86	0.99

CPS: clinical prediction score; PPV: positive predictive value; % VL done: percentage of patients that would undergo targeted VL testing; LHR: likelihood ratio.

## Discussion

We developed four simplified CPSs, of which three performed relatively well and have a number of advantages. Compared to the original CPS, no regular laboratory monitoring while on ART is required. Moreover, with a choice of CPS, programs can decide which is most appropriate and feasible to apply in their program settings. Performing hemoglobin measurement as part of an ART monitoring strategy can be cumbersome in many settings, comes with an additional cost and is not required in the latest WHO guidelines. Moreover, the added value in terms of diagnostic performance was limited, especially at lower cut-off values. CPS2 and CPS3 seem most useful and practical. CPS2 could be used when a baseline CD4 cell count is available, while CPS3 would apply when patients have initiated ART without CD4 guidance. With only a limited amount of key clinical information and minimal calculation required, these CPSs are also substantially simpler and possibly more adapted for use by non-physicians.

Some resource-constrained countries predominantly use clinical monitoring to detect treatment failure and CD4 cell counts are not always regularly done while on ART, especially in remote areas. However, we found that, in line with previous studies, CPS4 - relying on clinical information only - performed poorly [2]. Adding a CD4 cell count test ‘on the spot’ would substantially improve the performance of the algorithm without requiring routine CD4 monitoring. Current CD4 count was also found to be a strong predictor in a Ugandan scoring system [9]. Of interest, the same CD4 count cut-off (250 cells/µL) was identified in a recent study using data from the DART trial [21]. Pending the development of an affordable and widely available point of care VL test, use of these CPSs combined with targeted VL testing should be considered. If CD4 count testing can be done ‘on site’ or with a point of care test [22], application of the CPS could be done within one day. If not, patients might need two visits to apply the CPS. However, this creates an additional burden for both patients and health care staff, and increases chances for delays and missed appointments.

Further study is required to determine at what time points during ART these scores should be applied, and in which different health care settings and patient populations. Moreover, these algorithms were designed for patients on ART for at least one year. One approach could consist of a routine VL early after ART initiation (eg six months after ART initiation) followed by application of the score at predefined intervals, combined with targeted VL testing. Whereas applying the score every six months would equate to routine CD4 count monitoring, the current CPS would still have the advantage of not requiring interpretation of a trend of laboratory tests. While these simplifications would likely render them easier to apply and less error-prone compared to the original CPS, this is subject to further study.

A number of issues remain to be assessed before implementing these CPSs in routine care. Validation in other study populations and different settings should be conducted. Additional studies on the clinical utility and impact are warranted [23], [24]. The study population in this analysis consisted of patients on ART for several years. Possibly, the optimal ‘current CD4 cell count’ cut-off might differ in patient populations with short or very long treatment duration. Moreover, most patients in our cohort started ART with relatively advanced HIV disease. It remains to be assessed whether the performance of the CPSs would be different in patients starting early ART, as would occur within a test-and-treat strategy.

Additional limitations include the fact that treatment failure was based on a single VL measurement, in line with the previous derivation and validation study. Since a substantial proportion might have an undetectable VL on repeat measurement, only a proportion of those with virological failure in our study would be true treatment failures. On the other hand, the main purpose of targeted VL testing is early detection of active viral replication, since these patients need enhanced adherence counseling followed by a repeat VL. We did not evaluate whether individual opportunistic infections should be included as predictors in the CPS, but used ‘pooled’ WHO clinical staging groups. While this might be a more ‘crude’ approach, it has the advantage of being simpler. Finally, the failure rate in our study was clearly lower than what has been observed in most other programs. Consequently, positive predictive values will be higher and negative predictive values lower in settings with higher failure rates.

We acknowledge that the present algorithms are unlikely to be universally applicable and generalizable across a wide range of geographical regions, populations and health care settings. Derivation and validation of algorithms for local use (*eg* at the country or regional level) might be a reasonable alternative, and might also be valuable for a wider range of diagnostic tests or health care interventions. Targeted evaluation using evidence-based tools might be a rational way to optimize the use of often scarce resources available for health delivery in LMIC [2]. At the same time, such an approach also appreciates that patient populations are not homogeneous, but consist of different ‘risk groups’ with different needs and benefits of testing and other interventions. Especially as long as cheap point of care tests are lacking, such approaches merit further study.

In conclusion, we have developed three simplified CPSs for targeted VL testing that retained an acceptable diagnostic performance as long as ‘spot’ CD4 testing was included. They have the advantages of not requiring regular CD4 cell count or hemoglobin testing, or complete and reliable data collection while on ART, or the need to interpret trends in laboratory tests. They should, therefore, be less prone to error and more readily adopted in field conditions. Their use would enable more accurate targeted VL testing in contexts where it is too expensive to be done routinely. We suggest that they be tested and tailored to the other health care settings.

## References

[1] World Health Organization (2011) GLOBAL HIV/AIDS RESPONSE: Epidemic update and health sector progress towards Universal Access: Progress report, November 2011. Geneva, Switzerland.

[2] Lynen L, van Griensven J, Elliott J (2010) Monitoring for treatment failure in patients on first-line antiretroviral treatment in resource-constrained settings. Curr Opin HIV AIDS 5: 1–5. 20046141 10.1097/COH.0b013e3283333762

[3] World Health Organization (2013) Consolidated guidelines on the use of antiretroviral drugs for treating and preventing HIV infection. Recommendations for a public health approach. Geneva, Switzerland. 24716260

[4] Reid SD, Fidler SJ, Cooke GS (2013) Tracking the progress of HIV: the impact of point-of-care tests on antiretroviral therapy. Clin Epidemiol 5: 387–396. 24124392 10.2147/CLEP.S37069 PMC3794838

[5] Kumarasamy N, Krishnan S (2013) Beyond first-line HIV treatment regimens: the current state of antiretroviral regimens, viral load monitoring, and resistance testing in resource-limited settings. Curr Opin HIV AIDS 8: 586–590. 24100872 10.1097/COH.0000000000000004

[6] World Health Organization (2006) Antiretroviral therapy for HIV infection in adults and adolescents: Recommendations for a public health approach: 2006 Revision. Geneva, Switzerland. 23741771

[7] Rutherford GW, Anglemyer AT, Easterbrook PJ, Horvath T, Vitoria M, et al.. (2013) Predicting treatment failure in adults and children on antiretroviral therapy: systematic review of the performance characteristics of the 2010 World Health Organization criteria for virologic failure. 10.1097/QAD.0000000000000236 24849476

[8] Lynen L, An S, Koole O, Thai S, Ros S, et al (2009) An algorithm to optimize viral load testing in HIV-positive patients with suspected first-line antiretroviral therapy failure in Cambodia. J Acquir Immune Defic Syndr 52: 40–48. 19550349 10.1097/QAI.0b013e3181af6705

[9] Abouyannis M, Menten J, Kiragga A, Lynen L, Robertson G, et al (2011) Development and validation of systems for rational use of viral load testing in adults receiving first-line ART in sub-Saharan Africa. AIDS 25: 1627–1635. 21673555 10.1097/QAD.0b013e328349a414 PMC3725464

[10] Phan V, Thai S, Koole O, Menten J, Meheus F, et al.. (2013) Validation of a clinical prediction score to target viral load testing in adults with suspected first line treatment failure in resource-constrained settings. J Acquir Immune Defic Syndr. 10.1097/QAI.0b013e318285d28c 23334504

[11] Labhardt ND, Lejone T, Setoko M, Poka M, Ehmer J, et al (2012) A clinical prediction score in addition to WHO criteria for anti-retroviral treatment failure in resource-limited settings–experience from Lesotho. PLoS One 7: e47937. 23118910 10.1371/journal.pone.0047937 PMC3485299

[12] Colebunders R, Moses KR, Laurence J, Shihab HM, Semitala F, et al (2006) A new model to monitor the virological efficacy of antiretroviral treatment in resource-poor countries. Lancet Infect Dis 6: 53–59. 16377535 10.1016/S1473-3099(05)70327-3

[13] Meya D, Spacek LA, Tibenderana H, John L, Namugga I, et al (2009) Development and evaluation of a clinical algorithm to monitor patients on antiretrovirals in resource-limited settings using adherence, clinical and CD4 cell count criteria. J Int AIDS Soc 12: 3. 10.1186/1758-2652-12-3 PMC2664320 19261189

[14] World Health Organization (2010) Antiretroviral therapy for HIV infection in adults and adolescents, Recommendations for a public health approach, 2010 Revision. Geneva, Switzerland. 23741771

[15] Choun K, Thai S, Pe R, Lorent N, Lynen L, et al (2013) Incidence and risk factors for tuberculosis in HIV-infected patients while on antiretroviral treatment in Cambodia. Trans R Soc Trop Med Hyg 107: 235–42. 23324313 10.1093/trstmh/trt001

[16] Thai S, Koole O, Un P, Ros S, De MP, et al (2009) Five-year experience with scaling-up access to antiretroviral treatment in an HIV care programme in Cambodia. Trop Med Int Health 14: 1048–1058. 19573140 10.1111/j.1365-3156.2009.02334.x

[17] van Griensven J, Thai S (2011) Predictors of immune recovery and the association with late mortality while on antiretroviral treatment in Cambodia. Trans R Soc Trop Med Hyg 105: 694–703. 21962614 10.1016/j.trstmh.2011.08.007

[18] Seymour DG, Green M, Vaz FG (1990) Making better decisions: construction of clinical scoring systems by the Spiegelhalter-Knill-Jones approach. BMJ 300: 223–226. 2106929 10.1136/bmj.300.6719.223 PMC1662045

[19] Spiegelhalter DJ (1985) Statistical methodology for evaluating gastrointestinal symptoms. Clin Gastroenterol 14: 489–515. 4064353

[20] Berkley JA, Ross A, Mwangi I, Osier FH, Mohammed M, et al (2003) Prognostic indicators of early and late death in children admitted to district hospital in Kenya: cohort study. BMJ 326: 361–366. 12586667 10.1136/bmj.326.7385.361 PMC148891

[21] Gilks CF, Walker AS, Munderi P, Kityo C, Reid A, et al (2013) A single CD4 test with 250 cells/mm3 threshold predicts viral suppression in HIV-infected adults failing first-line therapy by clinical criteria. PLoS One 8: e57580. 23437399 10.1371/journal.pone.0057580 PMC3578828

[22] Sukapirom K, Onlamoon N, Thepthai C, Polsrila K, Tassaneetrithep B, et al (2011) Performance evaluation of the Alere PIMA CD4 test for monitoring HIV-infected individuals in resource-constrained settings. J Acquir Immune Defic Syndr 58: 141–147. 21709568 10.1097/QAI.0b013e31822866a2

[23] Holmberg L, Vickers A (2013) Evaluation of prediction models for decision-making: beyond calibration and discrimination. PLoS Med 10: e1001491. 23935462 10.1371/journal.pmed.1001491 PMC3728013

[24] McGinn TG, Guyatt GH, Wyer PC, Naylor CD, Stiell IG, et al (2000) Users’ guides to the medical literature: XXII: how to use articles about clinical decision rules. Evidence-Based Medicine Working Group. JAMA 284: 79–84. 10872017 10.1001/jama.284.1.79

